# ADVANCING HEALTHY AND SOCIAL LIVES BEYOND LATE-LIFE FUNCTIONAL LIMITATIONS: THE ROLE OF CONGREGATE SENIOR HOUSING

**DOI:** 10.1093/geroni/igae098.0293

**Published:** 2024-12-31

**Authors:** Byeongju Ryu, BoRin Kim, Sojung Park, Jihye Baek

**Affiliations:** Washington University in St. Louis, St. Louis, Missouri, United States; University of New Hampshire, Durham, New Hampshire, United States; Washington University in St. Louis, St. Louis, Missouri, United States; Washington University in St. Louis, St. Louis, Missouri, United States

## Abstract

Older adults with functional limitations often move into congregate senior housing (e.g., retirement communities and independent living) to maintain social and healthy lifestyles. Research showed mixed findings: some indicated improved social and physical activity from proximity to neighbors and organized activities in housing, while others highlighted negative outcomes such as disruption of existing social networks by relocation and social exclusion of frailer individuals within senior housing. Drawing on the National Health and Aging Trends Study Disability Conceptual Framework, this study examined the association between living in congregate senior housing and social/physical activity participation and whether perceived social cohesion moderates the association. We employed weighted logistic regression models to analyze NHATS Round 9 participants (n=2918) who reported at least one limitation with self-care (e.g., eating), mobility (e.g., getting around inside), or household activity (e.g., preparing meals), excluding those with dementia or in long-term care settings. Our findings indicated that residing in congregate senior housing was positively associated with participation in clubs/organized events [OR=1.43, p<.05], volunteering [OR=1.59, p<.05], and walking for exercise [OR=1.72, p<.01]. Housing types showed no differential influence on the residents’ activities, including visiting friends/family, attending religious services, and going out for enjoyment. We also found that senior housing residents were more likely to participate in organized events when they had stronger perceived social cohesion [OR=1.27, p<.05]. These findings suggest that congregate senior housing potentially enhances access to certain activities for those with functional limitations and that fostering perceived social cohesion is vital for organized activities in senior housing.

